# Potential impact of alternative vaccination strategies on COVID‐19 cases, hospitalization, and mortality in Japan during 2021–2022

**DOI:** 10.1002/jgf2.493

**Published:** 2021-08-26

**Authors:** Yasuharu Tokuda, Toshikazu Kuniya, Kenji Shibuya

**Affiliations:** ^1^ Muribushi Okinawa Center for Teaching Hospitals Okinawa Japan; ^2^ Graduate School of System Informatics Kobe University Kobe Japan; ^3^ Soma City COVID Vaccination Medical Center Fukushima Japan

Countries with rapid vaccination have reduced the COVID‐19 morbidity and mortality substantially.^1^ Japan has been ramping up the vaccination coverage, but the pace is stagnated because of the vaccine shortage at the time of the largest surge of daily cases since the pandemic began.^1^, ^2^ There is a growing number of cases and hospital admissions among unvaccinated adults in the age bracket of 30–50. This shortage is primarily driven by a general guideline of two doses with a three‐week interval for the public vaccination program. There are potentially enough vaccines for the first shot among adults,^3^ but the current schedule does not allow the vaccination of younger adults (aged less than 65) unless two doses of vaccination for the elderly are completed. The government has also introduced workplace mass vaccination program targeting those aged less than 65, which is also pending or canceled because of a shortage.

Recent studies suggest that vaccination intervals could be extended while waiting for the arrival of additional vaccines (interval extension strategy) without compromising expected immunity.^4^ One of the major concerns about this strategy is that there is a trade‐off between wider vaccine coverage with a first dose and limited effectiveness particularly among the elderly population when we would extend the interval. Alternatively, half a standard dose of the vaccine (half a dose strategy) could also be used without compromising the clinical immune effects.^5^ The problem is that this is not officially recommended by the manufactures and regulatory authority. Here, we present simulated results of the COVID‐19 epidemic projections using these two alternative vaccine strategies in Japan based on cases, hospitalization, and morality from January 2020 until August 2021. The model mainly considered the spread of Delta variant with greater infectiousness; we assumed the basic reproductive number of 8 (greater than 2 times as infectious as previous variants) based on the data from the US Center for Disease Control and Prevention.^6^

A SEIR compartmental model with vaccination was used for the curve fitting by updating the estimation per wave.^7^, ^8^ The infection rate was estimated for each period by fitting the model solution to the data before August 12, 2021. On the estimation, we used the data of daily vaccine doses after April 12, 2021.^9^, ^10^ More precisely, we divided the population into elderly and younger populations after that date and assumed that if a susceptible individual is vaccinated, then he/she enters the first vaccinated class. In addition, we considered the second vaccinated class and assumed that the infection prevention effects by the first and second vaccinations are 30% and 60%, respectively, and the onset (symptomatic development) prevention effects by the first and second vaccinations are 40% and 80%, respectively. We, moreover, assumed that the immunity induced by the second vaccination will be decreased to 80% after 6 months and to 50% eventually.

Assumptions for prediction after August 13, 2021, include the following: If the number of new infections exceeds 10,000 during the period of infection spread, restrictions (i.e., a state of emergency—a Japanese version of mild lockdown) will be declared. Restrictions will be lifted when the number of new infections falls below 1000 during the period of infection control. We assumed that the number of daily vaccines is 700,000 and allocate them to (i) elderly and younger people and (ii) the first and second vaccinations. The baseline ratios of both elderly and younger people were 5:5, and we discuss each scenario by changing these ratios.

Figure 1A shows the results of changing the ratio of vaccine allocation between the first and second doses based on the interval extension strategy starting from September 2021. The number of infected cases would be decreased when more vaccine was allocated to the first dose. A larger number of the young people receiving the first dose seemed to have a greater effect.

**FIGURE 1:**
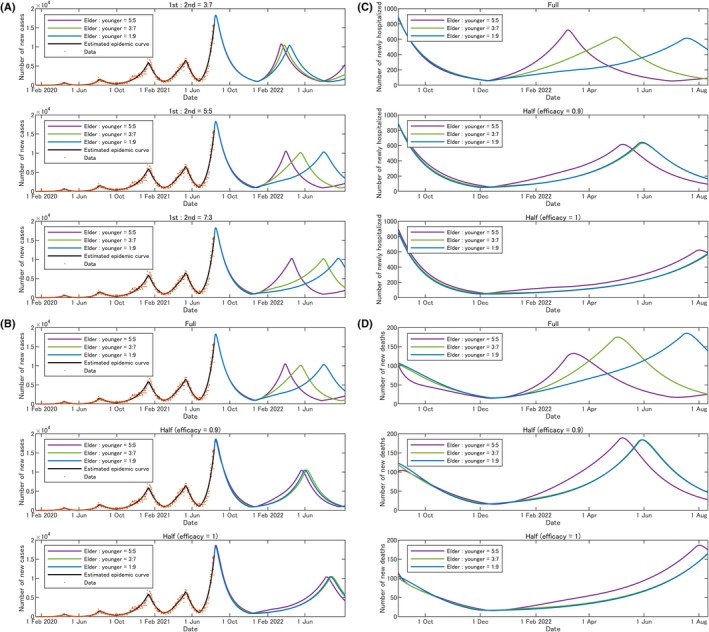
(A) Interval extension strategy. Changing the ratio of vaccine allocation between the first and second doses based on the interval extension strategy starting from September 2021. The simulation assumed three different distribution ratios for the elderly and young: The distribution ratio is 5:5 (elderly:young) in purple, 3:7 in green, and 1:9 in blue curves. (B) Half a dose strategy. The simulation employed half a standard dose of vaccination and thus doubled the pace from the daily pace for the entire population of Japan with vaccination with slightly lower (efficacy proportion = 0.9) or the similar (efficacy proportion = 1) effects, starting from September 2021. The simulation assumed three different distribution ratios for the elderly and young: The distribution ratio is 5:5 (elderly:young) in purple, 3:7 in green, and 1:9 in blue curves. (C) Expected hospitalization cases by half a dose strategy. The simulation employed half a standard dose of vaccination similarly to that of B. (D) Expected death cases by half a dose strategy. The simulation employed half a standard dose of vaccination similarly to that of B

Next, also starting from September 2021, the simulation employed half a standard dose of vaccination (half a dose strategy) and thus doubled the roll‐out of vaccination with slightly lower (efficacy proportion = 0.9) or the similar (efficacy proportion = 1) vaccine effects. During the doubled vaccination pace, the simulation assumed three different distribution ratios for the elderly and young. Figure 1B shows the curves by the ratios: The distribution ratio is 5:5 (elderly:young) in purple curve. However, the number of infected cases seems to be decreased when distribution ratios of 3:7 (green) and 1:9 (blue) are used. These effects are likely to reflect that the vaccinated proportion has been already high among the elderly and only allocating more vaccine, such as 50% of the available vaccines, to the young could change the results. Even in the full‐dose schedule (the uppermost graph of Figure 1B), such allocation could reduce cases. When half a dose strategy would be employed, a surge of hospitalization and mortality could be delayed (Figure 1C,D). The peak cases of both hospitalization and mortality seem to be like that of the current strategy because of the decreased immunity of the vaccines. However, with a delayed peak provided by half a dose strategy, we could gain precious time interval to prepare and conduct a booster vaccine program to reduce the peaks.

As of August 2021, the delta variant of concern is already spreading throughout Japan, while available vaccines are limited. Flexible vaccination strategies should be considered for delaying infection peaks to obtain time interval to reduce the number of cases, hospitalization, and mortality. One‐dose vaccination (i.e., interval extension strategy) for a wider population and/or low‐dose vaccination (i.e., half a dose strategy) for many could resolve the current vaccine shortage and suppress a surge requiring lockdown. The rapid implementation of these potentially life‐saving strategies should be considered for unprecedented phase that are coming for next several months.

## CONFLICT OF INTEREST

The authors have stated explicitly that there are no conflicts of interest in connection with this article.
